# Hemostatic Interventions and All-Cause Mortality in Hemodynamically Unstable Pelvic Fractures: A Systematic Review and Meta-Analysis

**DOI:** 10.1155/2024/6397444

**Published:** 2024-08-26

**Authors:** XuWen Zheng, MaoBing Chen, Yi Zhuang, Jin Xu, Liang Zhao, YongJun Qian, WenMing Shen, Ying Chu

**Affiliations:** ^1^ Truama Center Wujin People's Hospital Affiliated with Jiangsu University and Wujin Clinical College of Xuzhou Medical University, Changzhou 213017, China; ^2^ Wujin Institute of Molecular Diagnostics and Precision Cancer Medicine of Jiangsu University, Changzhou 213017, China

## Abstract

**Objective:**

To conduct a systematic review and meta-analysis of the all-cause mortality associated with the most commonly used hemostatic treatments in patients with hemodynamically unstable pelvic fractures.

**Methods:**

Up to April 30, 2023, we searched PubMed, Embase, Web of Science, and Cochrane, including the references to qualified papers. A meta-analysis was performed on studies that reported odds ratios (ORs) or the number of events needed to calculate them. The PROSPERO registration number was CRD42023421137.

**Results:**

Of the 3452 titles identified in our original search, 29 met our criteria. Extraperitoneal packing (EPP) (OR = 0.626 and 95% CI = 0.413–0.949), external fixation (EF) (OR = 0.649 and 95% CI = 0.518–0.814), and arterial embolism (AE) (OR = 0.459 and 95% CI = 0.291–0.724) were associated with decreased mortality. Resuscitative endovascular balloon occlusion of the aorta (REBOA) (OR = 2.824 and 95% CI = 1.594–5.005) was associated with increased mortality. A random effect model meta-analysis of eight articles showed no difference in mortality between patients with AE and patients with EPP for the initial treatments for controlling blood loss (OR = 0.910 and 95% CI = 0.623–1.328).

**Conclusion:**

This meta-analysis collectively suggested EF, AE, or EPP as life-saving procedures for patients with hemodynamically unstable pelvic fractures.

## 1. Introduction

Pelvic fractures represent 3% of all skeletal injuries and are predominantly observed in young adults [1, 2]. These fractures are highly lethal due to the rapid loss of blood and the severity of associated injuries [3]. Patients with these injuries often experience major complications, such as cardiac arrest, infectious diseases, respiratory distress, and venous thromboembolism, which significantly contribute to their high mortality rates [4]. Various studies have explored the efficacy of hemostatic treatments such as arterial embolism (AE), resuscitative endovascular balloon occlusion of the aorta (REBOA), extraperitoneal packing (EPP), and external fixation (EF) in reducing mortality in patients with hemodynamically unstable pelvic fractures [5–7]. However, these studies have yielded mixed results, with some reporting significant reductions in mortality and others showing limited or no benefits [8–10].

This inconsistency in the research findings underscores the need for a systematic review and meta-analysis to assess and synthesize the available evidence. By focusing exclusively on hemodynamically unstable fractures, this study aims to clarify the effectiveness of the mentioned interventions. It systematically searches the literature without language restrictions to include a comprehensive range of studies and uses rigorous meta-analytical techniques to evaluate the impact of these treatments on mortality rates. This analysis is crucial for improving clinical decision-making and outcomes in trauma care, particularly in emergency settings where rapid and effective intervention is critical. The findings of this meta-analysis are expected to provide valuable insights that could guide the development of treatment protocols and influence clinical practices worldwide, ultimately improving survival rates and quality of care for victims of severe pelvic trauma.

## 2. Materials and Methods

This protocol has been registered in the International Prospective Register of Systematic Reviews (PROSPERO) under registration number CRD42023421137. This study did not require ethical approval because it used data that were already in the public domain. The PRISMA checklist is shown in Supplementary 1.

### 2.1. Search Strategy

Electronic searches were conducted in PubMed, Embase, Web of Science, and Cochrane, utilizing a full list of MeSH headings and text words from prior reviews and search tools in Ovid, PubMed, and Embase. We searched for published articles up to April 30, 2023, with no language restrictions.  Supplementary 2 describes the search strategy we utilized.

### 2.2. Study Selection

All studies that met the following criteria were included: (1) were adult patients; (2) had pelvic fractures caused by blunt pelvic injury; (3) were hemodynamically unstable or in hypovolemic shock when arriving at the emergency department; (4) examined the relationship between hemostatic interventions (AE, REBOA, EF, and EPP) and mortality in hemodynamically unstable pelvic fractures; and (5) provided odds ratios (ORs) and 95% confidence intervals (CIs) or the number of events that can calculate them. Studies that met the abovementioned inclusion criteria were excluded if they also met the following criteria: (1) were duplicate articles or data, (2) were nonhuman studies, (3) were review articles or letters, (4) had insufficient data or information to calculate ORs, or (5) the sample size was less than 20. Based on the predetermined selection criteria, all studies retrieved from the database were assessed independently by two researchers. Additionally, a third investigator resolved disagreements through discussion or consultation.

### 2.3. Data Extraction

For each eligible study, two reviewers retrieved the following data independently: first author's name, country, sample size, study design, publication year, demographic factors (e.g., sex and age), outcome (mortality), exposure (e.g., AE, REBOA, PP, and EF), and adjusted odds ratios. Any conflicts were settled through consensus.

### 2.4. Methodological Quality Assessment

The Newcastle‒Ottawa scale (NOS), which is commonly used for assessing the quality of nonrandomized studies in a meta-analysis [11], was used for quality assessment. The NOS included eight items, which were grouped into the following three categories: (1) study group selection, (2) group comparability, and (3) outcome of interest. A score of 1 was given for each item in each study. High-scoring studies were considered good reports. Two authors evaluated the scores, and any inconsistencies were resolved through discussion between the two evaluating authors. A score greater than 7 suggested that there was a low risk of bias.

### 2.5. Statistical Analysis

STATA version 17 was used to examine the combined associations by computing pooled odds ratios (ORs) and 95% confidence interval (CI). The *Q* test was employed to examine effect size heterogeneity. The *I*^2^ statistic was used to calculate the fraction of total variance that may be attributed to study heterogeneity [12]. The statistical results ranged from 0% to 100% (*I*^2^ = 0–25% for no heterogeneity, *I*^2^ = 25–50% for mild heterogeneity, *I*^2^ = 50–75% for moderate heterogeneity, and *I*^2^ = 75–100% for large heterogeneity). If there was mild or moderate heterogeneity, the random effects model was utilized, and if there was moderate heterogeneity, a metaregression was performed to investigate the sources of heterogeneity. If there was high heterogeneity, a meta-analysis was not performed. Otherwise, if *I*^2^ was less than 25%, the fixed effects model was utilized. Random effects models are widely used to account for heterogeneity among study results in meta-analyses, while fixed effects models assume that the effect size is constant across all studies. These models incorporate a between-study variance component, allowing for more realistic estimates of overall treatment effects [13, 14]. We also examined for possible publication bias by looking at the funnel plots of the main outcome and using the Egger weighted linear regression test to determine whether the funnel plots were symmetrical [15]. This test examines the association between the observed effect sizes and their standard errors using a linear regression approach. A significant intercept suggests the presence of publication bias [15]. If the funnel was asymmetric, a trim-and-fill method was used to assess and compensate for publication bias [16].

## 3. Results

### 3.1. Search Results and Study Inclusion Criteria

**Figure 1:**
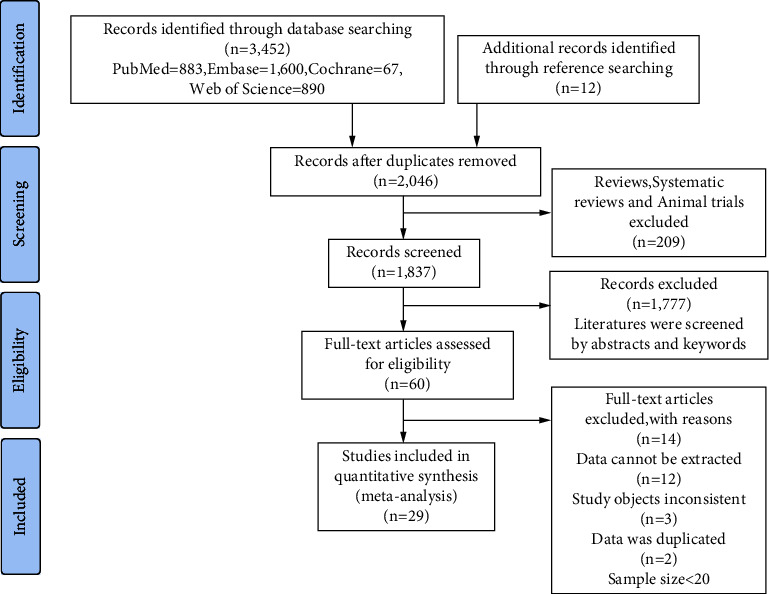
Flow diagram of the study selection process based on the criteria of preferred reporting items for systematic reviews and meta-analyses.

Following the initial search, 3440 documents were obtained from four databases, and another 12 records were identified by reviewing reference citations. We eliminated 1406 studies due to duplication. Then, 209 papers were omitted because they were reviews or animal trials. After reviewing the titles and abstracts, 1777 articles were eliminated. Sixty studies were downloaded and evaluated for eligibility after reviewing the complete text, and 29 articles were included in this meta-analysis. The detailed selection process is depicted in Figure 1.

### 3.2. Study Characteristics

Table 1 shows a summary of the main features of the 29 studies that were included. There were eight studies that evaluated the use of AE, eleven studies that evaluated EPP, eight studies that evaluated EF, six studies that evaluated REBOA in pelvic fractures with unstable hemodynamics, and eight studies that compared AE to EPP. All the studies were published between 2002 and 2023 and included results from Asia, Europe, the United States, and Australia. The investigations included 25 retrospective cohort studies, 3 prospective cohort studies, and 1 quasirandomized trial employing the NOS. Using the NOS score, 18, 6, and 5 studies received 9, 8, and 7 points, respectively.

#### 3.2.1. AE

**Figure 2:**
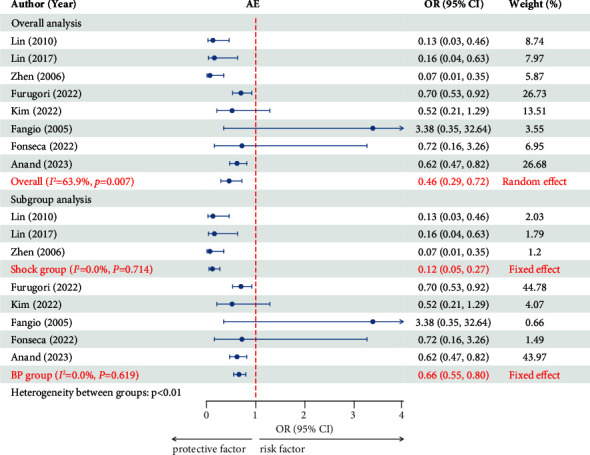
Forest plot showing the effect of AE on mortality. AE, arterial embolism; OR, odds ratio; CI, confidential interval; BP, blood pressure.

The data were pooled from eight trials involving 4607 participants [4, 18, 21, 32–36]. The eight studies showed moderate heterogeneity (50% < *I*^2^ = 63.9% < 75%, *p*=0.007). For the eight studies, a random effect was chosen, and AE was found to be a protective factor against death in patients with hemodynamically unstable pelvic fractures (OR = 0.459, 95% CI = 0.291–0.724, *p*=0.01) (Figure 2). A symmetric funnel plot was constructed for publication bias (Supplementary 3). Egger's test was also used (*p*=0.184), which demonstrated that there was no publication bias in this study. The heterogeneity may have been produced by the varied criteria for hemodynamic instability; consequently, a meta-regression was chosen to determine the source of heterogeneity. The regression coefficient of the hemodynamic variables was *p*=0.01. This revealed that the criterion of hemodynamic instability had a major effect on the effect size and was the source of heterogeneity. The eight studies were subsequently separated into two subgroups based on different hemodynamic instability criteria, namely, the shock group (shock index >1.5) and the BP group (initial BP <90 mmHg). There was no intragroup heterogeneity in the shock group (*I*^2^ = 0, *p*=0.714) or the BP group (*I*^2^ = 0, *p*=0.619), but there was intergroup heterogeneity between the two subgroups (*p* < 0.01). A subgroup meta-analysis was performed based on the different hemodynamic instability criteria. The pooled data for the shock group revealed that AE was a protective factor against death in patients with hemodynamically unstable pelvic fractures (OR = 0.117, 95% CI = 0.051–0.267, and *p* < 0.01). The pooled results for the BP group also revealed that AE was a protective factor against death in patients with hemodynamically unstable pelvic fractures (OR = 0.661, 95% CI = 0.547–0.798, and *p* < 0.01) (Figure 2). A symmetric funnel plot (Supplementary 3) was constructed from the publication bias test results for the two subgroups. Egger tests were also carried out. There was no publication bias in the shock or BP groups (*p*=0.339 and *p*=0.463, respectively).

#### 3.2.2. REBOA

**Figure 3:**
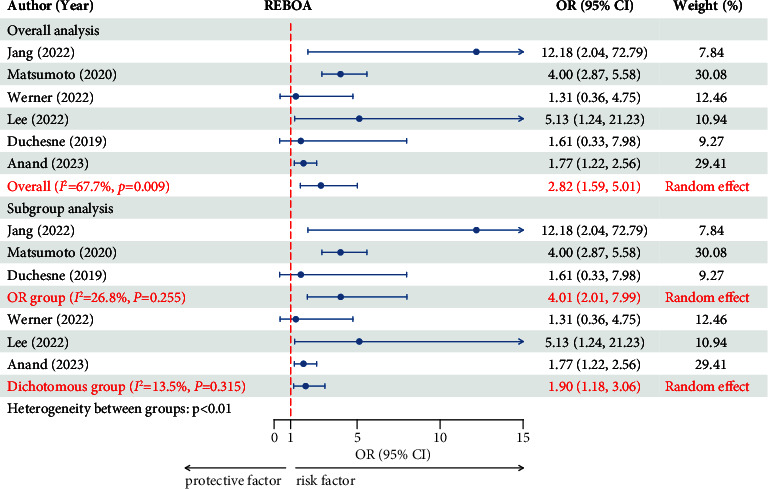
Forest plot showing the effect of REBOA on mortality. REBOA, resuscitative endovascular balloon occlusion of the aorta; OR, odds ratio; CI, confidential interval.

The data were pooled from 6 studies of 5165 patients [4, 9, 22, 24, 30, 31]. Moderate heterogeneity was found among the six studies (50% < *I*^2^ = 67.7% < 75%, *p*=0.009). A random effect was selected for the six studies. The combined results showed that REBOA was a risk factor for mortality in patients with hemodynamically unstable pelvic fractures (OR = 2.824, 95% CI = 1.594–5.005, *p* < 0.01) (Figure 3). The publication bias test yielded a symmetric funnel plot (Supplementary 3). Egger's test was also performed (*p*=0.911), indicating that there was no publication bias in this study. We found that three of the six studies reported ORs as effect sizes, and the other three reported dichotomous variable as effect sizes. The inconsistency of the effect sizes may have caused heterogeneity; thus, a meta-regression was selected to determine the source of heterogeneity. The regression coefficient was *p*=0.045, which indicated that the different effect sizes were the cause of heterogeneity. Subsequently, the six studies were divided into two subgroups, namely, the OR (reported ORs) group and the dichotomous group (reported dichotomous variable). The results showed mild intragroup heterogeneity in the OR group (*I*^2^ = 26.8% and *p*=0.255) and no intragroup heterogeneity in the dichotomous group (*I*^2^ = 13.5% and *p*=0.315), but intergroup heterogeneity was found between the two subgroups (*p* < 0.01). Subgroup meta-analysis was conducted based on the different types of effect sizes. For the OR group, the combined results showed that REBOA was a risk factor for mortality in patients with hemodynamically unstable pelvic fractures (OR = 4.009, 95% CI = 2.013–7.987, *p* < 0.01). For the Dichotomous group, the combined results also showed that REBOA was a risk factor for mortality in patients with hemodynamically unstable pelvic fractures (OR = 1.905, 95% CI = 1.184–3.063, and *p* < 0.01) (Figure 3). The publication bias test of the two subgroups showed a symmetric funnel plot (Supplementary 3). Egger's tests were also performed. No publication bias was found for the OR or dichotomous groups (*p*=0.96 and *p*=0.692, respectively).

#### 3.2.3. EF

**Figure 4:**
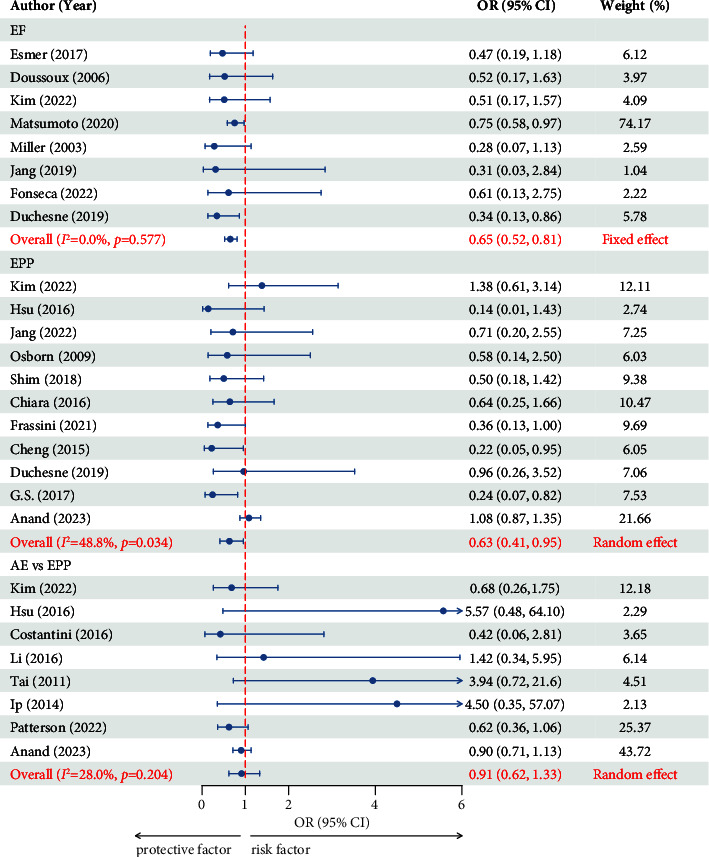
Forest plot showing the effect of EF, EPP, and AE vs. EPP on mortality. OR, odds ratio; CI, confidential interval; EF, external fixation; EPP, extraperitoneal packing; AE, arterial embolism.

The data were pooled from 8 studies of 3844 patients [9, 16–22]. No heterogeneity was found among the eight studies (*I*^2^ = 0 and *p*=0.577). A fixed effect was selected for the eight studies. The combined results showed that EF was a protective factor against mortality in patients with hemodynamically unstable pelvic fractures (OR = 0.649, 95% CI = 0.518–0.814, and *p* < 0.01) (Figure 4). The publication bias test yielded an asymmetric funnel plot (Supplementary 3). Egger's test was also performed (*p*=0.007), indicating publication bias in this study, and a trim-and-fill analysis was needed for bias correction. However, after two iterations, there was no indication of publication with the trim-and-fill method (no new studies were added) (Supplementary 3).

#### 3.2.4. EPP

The data were pooled from 11 studies of 2455 patients [3, 4, 18, 22–29]. Mild heterogeneity was found among the 11 studies (25% < *I*^2^ = 48.8% < 50% and *p*=0.034). A random effect was selected for the 11 studies. The combined results showed that the EPP was a protective factor against mortality in patients with hemodynamically unstable pelvic fractures (OR = 0.626, 95% CI = 0.413–0.949, and *p* < 0.05) (Figure 4). A publication bias test of the eleven articles in this study yielded an asymmetric funnel plot (Supplementary 3). Egger's test was also performed (*p*=0.005), indicating publication bias in this study, and a trim-and-fill analysis was needed for bias correction. However, after two iterations, there was no indication of publication with the trim-and-fill method (no new studies were added) (Supplementary 3).

#### 3.2.5. AE vs. EPP

The data were pooled from 8 studies of 1867 patients [4, 18, 23, 37–41]. Mild heterogeneity was found among the eight studies (25% < *I*^2^ = 28.0% < 50% and *p*=0.142). A random effect was selected for the eight studies. The combined results revealed no significant difference in mortality when AE or EPP were used as primary bleeding control measures for patients with hemodynamic instability (OR = 0.910, 95% CI = 0.623–1.328, and *p*=0.204) (Figure 4). The publication bias test yielded a symmetric funnel plot (Supplementary 3). Egger's test was also performed (*p*=0.283), indicating that there was no publication bias in this study.

## 4. Discussion

A thorough search strategy was implemented to acquire all relevant data. Despite the exclusion of numerous ostensibly pertinent articles from our meta-analysis due to unextractable data or inconsistent study objects, the quantitative findings of the included articles were generally consistent with the aggregated results. A comprehensive analysis of mortality resulting from hemodynamically unstable pelvic fractures was conducted by examining the AE, EPP, EF, and REBOA. The direct and dichotomous ORs were included in the derived effect sizes.

External pelvic fixation was recommended as an adjuvant for early bleeding control in hemodynamically unstable pelvic ring ruptures according to the WSES classification and guidelines [1]. Hu et al. showed that using an external fixator could lower the pelvic volume and blood clots while also putting direct pressure on bleeding arteries to help tamponade work [43]. Because the EPP does not work without enough counterpressure from the back of the pelvis, which means that unstable pelvic ring disruptions need to be fixed from the outside, the stable counterpressure that the EF provides is very important for the next step of extraperitoneal packing [44]. Furthermore, proper EF can minimize secondary damage during handling. For example, using an external fixator to stabilize the pelvis may prevent recurrent shocks to preexisting occluded arteries [45].

REBOA can be used as a “bridge” procedure in patients with abdominal pelvic or lower limb bleeding before a definitive operation. Zone 3 (infrarenal) REBOA can be optimal, especially for pelvic bleeding, because it involves a longer occlusion time, increased blood pressure, and reduced arterial bleeding associated with pelvic injury while preventing ischemic insult to visceral organs [46]. Several recent investigations, however, have shown that REBOA is related to a significant mortality rate in patients with hemodynamically unstable pelvic fractures. Jang et al. demonstrated that REBOA was not a risk factor for bleeding-related death while also confirming that it was a substantial risk factor for mortality [23]. Although REBOA can effectively control bleeding, one of the reasons for the increased mortality may be its consequences. Patients who received REBOA experienced problems such as limb ischemia, iatrogenic aortic dissection, acute renal injury, and rhabdomyolysis [47].

Our findings supported previous research showing that both AE and EPP could reduce mortality in patients with hemodynamically unstable pelvic fractures. According to the WSES guidelines, angioembolization is the best way to stop bleeding in patients whose retroperitoneal pelvic bleeding originates from an artery. Patients who experience hemodynamic instability due to pelvic fractures should be evaluated consistently for preperitoneal pelvic packing, particularly in medical facilities lacking angiography capabilities [1]. However, it is uncertain which comes first. McDonogh's meta-analysis of data from three studies involving 104 patients with EPP or AE as a primary bleeding control measure revealed no significant difference in mortality [11]. Despite the addition of five new trials, the data from eight studies, including 1867 participants in our study, revealed no significant difference in mortality. The need for rapid hemorrhage management in patients with continuous pelvic bleeding is currently undisputed although there is no agreement on a standard approach for treating patients with hemodynamically unstable pelvic fractures. The three primary sources of hemorrhaging in pelvic fractures are arterial damage, the surface of the fractured bones, and the pelvic venous plexus. Hemorrhaging following pelvic fractures is observed in approximately 90% of the cases involving veins and 10% of the cases involving arteries [48]. AE was utilized to control arterial bleeding. The main drawback of this procedure is its inability to control venous bleeding; hence, if the retroperitoneum perforates the abdominal cavity, delayed severe exsanguination may result. According to Li et al., patients with AE take longer to receive treatment after admission. Delaying embolization can be effective, but it increases the risk of death [37]. This could be attributed to either pelvic hemorrhaging or an erroneous selection made at the pivotal decision point between abdominal and pelvic hemorrhaging. External pelvic pressure is used to stop venous bleeding by directly pressing on the veins and arteries in the sacrum. The retroperitoneum is not breached, and hemostasis is achieved through direct pressure on the sacral plexus of veins and iliac vessels. The period from diagnosis to surgical intervention was greatly reduced. Identifying the predominant source of pelvic bleeding during the first resuscitation is difficult. In addition, arterial bleeding accounts for 10–15% of hemorrhages, while the primary causes of bleeding are the posterior pelvic venous plexus or fractured bone surfaces. Therefore, in patients with pelvic fractures who experience unstable hemodynamics, pelvic packing should be prioritized as the initial treatment option. If the patient continues to experience hemodynamic instability even after pelvic packing, it is important to examine the possibility of arterial bleeding, and angiographic embolization may be necessary.

Our findings suggested external fixation as a first step in patients with hemodynamically unstable pelvic fractures, with a pelvic bind or sheet as an alternative if no external fixation device is available. If pelvic hemorrhage is suspected, angiography can be used to determine the source of bleeding [49]. If the bleeding is of arterial origin, AE should be performed first; if it is of venous origin, EPP should be performed; if the source of bleeding is undetermined or if the hemorrhage comes from both arteries and veins, EPP should be utilized prior to AE. Although there is no significant difference in mortality between which approach comes first, EPP patients experience shorter preparation times and operation times, which could significantly reduce mortality and transfusion requirements in pelvic fracture patients [50]. If the patient continues to experience hemodynamic instability even after EPP, an AE should be performed promptly. REBOA was not considered unless extensive abdominal or lower extremity injuries occurred. Nevertheless, the particular hemostatic treatments can differ based on the characteristics and expertise of individual trauma centers. In facilities without access to interventional procedures, EPP emerges as the primary hemostatic intervention for patients experiencing hemodynamically unstable pelvic fractures subsequent to external fixation.

A key strength of this study is its comprehensive and systematic approach, as demonstrated by the extensive database search which included multiple databases like PubMed, Embase, Web of Science, and Cochrane up to April 2023. The meta-analysis incorporated studies that provided odds ratios or the number of events needed to calculate them, enhancing the reliability of the results. This systematic review and meta-analysis effectively synthesized diverse findings from 29 qualifying studies, applying rigorous meta-analytical techniques to ensure robust conclusions. This detailed analysis not only offers critical insights into the survival benefits of specific interventions but also supports improved clinical decision-making in trauma care, potentially influencing treatment protocols worldwide and contributing to better patient outcomes in emergency settings. This study, while comprehensive, has several limitations that warrant consideration. First, the included studies exhibit inherent biases due to their predominantly retrospective nature, which could affect the robustness of the findings. Notably, the heterogeneity observed across studies, as indicated by variable *I*^2^ values, suggests differences in study populations, intervention techniques, and outcome measures, which might influence the generalizability of the results. In addition, assumptions made during the meta-analysis, such as the choice of statistical models and the handling of missing data, might also impact the conclusions drawn. Such factors underscore the need for cautious interpretation of the pooled estimates and highlight the necessity for prospective, standardized trials to validate these findings and potentially guide clinical practice more reliably. Second, changes in follow-up time, which were not consistently reported, may limit the interpretability of the results. However, our findings were consistent across studies, relevant factors, follow-up durations, and continents, supporting the primary conclusions. Third, the complications of each measure, including the incidence and severity of complications, were not compared; these complications may have a significant impact on mortality in patients with hemodynamically unstable pelvic fractures. Our investigation ultimately identified publication bias in the analyses of EF and EPP. However, there was no evidence of additional data being included in the trim-and-fill method. These two analyses revealed either no heterogeneity or only mild heterogeneity, indicating the reliability of the pooled data.

## 5. Conclusion

This meta-analysis showed that EF, AE, or EPP, as life-saving procedures, could decrease mortality in patients with hemodynamically unstable pelvic fractures. In light of the findings, future research should focus on conducting prospective randomized controlled trials to address the limitations observed in retrospective studies and to validate the effectiveness of hemostatic interventions in hemodynamically unstable pelvic fractures. Specifically, research should aim to standardize intervention protocols and outcome measures, thereby reducing heterogeneity and improving the applicability of results. Emergency and trauma care protocols should integrate these findings to enhance decision-making processes, potentially improving survival outcomes. By advancing research and refining clinical practices based on solid evidence, healthcare providers can better address the complexities and challenges associated with managing severe pelvic injuries.

## Figures and Tables

**Table 1:** Main characteristics of the included studies.

Author (year)	Country	Study design	Definition of hemodynamically unstable	Sample	Intervention	Death/survival	Age, mean ± SD, y	Sex (male%)	NOS
						(OR for mortality)			
Lin (2010) [17]	China	Retrospective	Hypovolemic shock (shock index 1.7–3.0)	92	AE	3/36	35.3 ± 9.5	67	9
					Non-AE	21/32	37.2 ± 5.7	81	

Lin (2017) [18]	China	Retrospective	Hypovolemic shock (shock index 1.7–3.0)	65	AE	3/29	38.1 ± 4.5	78	9
					Non-AE	13/20	39.2 ± 5.7	79	

Zheng (2006) [19]	China	Retrospective	Hypovolemic shock (shock index 1.5–3.0)	68	AE	2/46	34.0 ± 8.5	78	9
					Non-AE	8/12	37.5 ± 9.5		

Esmer (2017) [20]	Germany	Retrospective	Systolic BP <100 mmHg and shock index >1	104	EF	9/38	40.2 ± 15	69	9
					Non-EF	19/38	49.2 ± 23.2		

Doussoux (2006) [21]	Spain	Retrospective	Systolic BP <90 mmHg or transfusion needs higher than 2 units of red cells in first 12 h	79	EF	7/40	38.8 ± 15.6	70	9
					Non-EF	8/24			

Furugori (2022) [22]	Japan	Retrospective	Systolic BP <90 mmHg	2806	AE	0.7 (0.53–0.92)	NA	NA	9

Hsu (2016) [23]	Australia	Prospective	Systolic BP <90 mmHg and/or initial base deficit >5	24	AE	3/7	60.3 ± 23.5	83	9
					EPP	1/13	49.9 ± 17.5		

Jang (2022) [24]	Korea	Retrospective	Systolic BP <90 mmHg or serum lactate level of ≥2 mmol/L	157	EPP	0.711 (0.198–2.551)	59.3 ± 17.3	68	8
					REBOA	12.183 (2.039–72.801)			

Costantini (2016) [25]	United States	Prospective	Systolic BP <90 mmHg or heart rate >120 beats per minute or base deficit >6	23	AE	5/12	NA	NA	7
					EPP	3/3			

Osborn (2009) [26]	United States	Prospective	Persistent systolic BP <90 mmHg (after receiving 2000 ml of intravenous crystalloid)	40	EPP	4/16	37.9 ± 18.9	NA	8
					Non-EPP	6/14	39.5 ± 17.4		

Fangio (2005) [27]	France	Retrospective	Systolic BP <90 mmHg after an additional infusion of normal saline (500 mL) and a continuous infusion of dopamine	32	AE	9/16	39 ± 17	63	7
					Non-AE	1/6	30 ± 5		

Shim (2018) [3]	Korea	Retrospective	Persistent systolic BP <90 mmHg despite the loading of two units of packed red cells	58	EPP	12/18	62.5 ± 14.4	67	9
					Non-EPP	16/12	57.0 ± 22.8	57	

Matsumoto (2020) [6]	Japan	Retrospective	Systolic BP <90 mmHg or heart rate >120 beats/min at admission	3149	EF	0.75 (058–0.98)	53.5 ± 21.4	58	9
					REBOA	4 (2.87–5.58)			

Ha et al. (2017) [28]	Korea	Retrospective	Hemorrhagic shock	53	EPP	5/23	NA	NA	7
					Non-EPP	12/13			

Chiara (2016) [29]	Italy	Retrospective	Persistent systolic BP <90 mmHg despite pelvic binder and ≥2 L of intravenous crystalloids and transfusion of ≥2 units of RBCs	78	EPP	10/20	55.3 ± 21.8	57	9
					Non-EPP	21/27	48.5 ± 20.8	73	

Frassini (2021) [30]	Italy	Retrospective	Systolic BP <90 mmHg despite pelvic binder and 1 L of intravenous crystalloids and transfusion of ≥2 units of red cells	74	EPP	8/16	NA	60	9
					Non-EPP	29/21		73	

Miller (2003) [31]	United States	Retrospective	Hypotension	35	EF	6/10	46 ± 51	80	7
					Non-EF	13/6			
Cheng (2015) [32]	Hong Kong, China	Retrospective	Systolic BP <90 mmHg upon arrival at AED or at any time during hospital stay after infusion of 2 L of crystalloids	199	EPP	0.223 (0.052–0.947)	NA	56	9

Jang (2019) [33]	Korea	Retrospective	Persistent systolic BP <90 mmHg despite 2 L crystalloid loading and transfusion of 2 units of red cells	50	EF	1/6	59.6 ± 18.6	56	9
					Non-EF	15/28			

Werner (2022) [34]	United States	Retrospective	Persistent systolic BP <90 mmHg after initial transfusion of 2 units of red cells in the emergency department	78	REBOA	5/26	50 ± 17.6	74	9
					Non-REBOA	6/41	49 ± 17.6	68	

Lee (2022) [35]	Korea	Retrospective	Systolic BP <90 mmHg during the initial resuscitation despite the transfusion of 2 units of packed RBCs	106	REBOA	7/3	52.3 ± 19.8	50	9
					Non-REBOA	30/66			

Fonseca (2022) [36]	Brazil	Retrospective	Systolic BP <90 mmHg and BE >−5 mmol/L	51	EF	23/19	NA	75	8
					Non-EF	6/3			
					AE	4/4			
					Non-AE	25/18			

Li (2016) [37]	China	Quasi-randomized	Systolic BP <90 mmHg after administration of 4 units of red cells	56	AE	5/22	40 ± 9	59	8
					EPP	4/25	43 ± 13	56	

Tai (2011) [38]	Hong Kong, China	Retrospective	Persistent systolic BP ≤90 mmHg after receiving ≥2000 mL intravenous crystalloid	24	AE	9/4	44.8 ± 24.7	63	9
					EPP	4/7	51.2 ± 19.0		

Patterson (2022) [39]	United States	Retrospective	Systolic BP <90 mmHg	366	AE	27/156	37.4 ± 2.1	74	8
					EPP	40/143	37.3 ± 2.0	72	

Ip (2014) [40]	Hong Kong, China	Retrospective	Persistent systolic BP ≤90 mmHg after receiving ≥2000 mL intravenous crystalloid	29	AE	2/1	42.0 ± 11.3	100	7
					EPP	8/18	46.3 ± 20.2	42	

Duchesne (2019) [41]	United States	Retrospective	Systolic BP <90 mmHg, heart rate >120 beats per minute, or BE >−5	279	REBOA	1.613 (0.326–7.971)	40 ± 14	62	9
					EPP	0.959 (0.261–3.522)			

Anand (2023) [4]	United States	Retrospective	Received 4 or more units of packed RBCs within 4 hours of presentation, and underwent at least 1 pelvic fracture hemorrhage control intervention. Lowest SBP <90 mmHg	1396	AE	0.62 (0.47–0.82)	47 ± 19	70	9
					EPP	243/416			
					Non-EPP	258/479			
					REBOA	61/65			
					Non-REBOA	440/830			
				1270	AE	218/434			
					EPP	222/396			

Kim (2022) [42]	Korea	Retrospective	SBP <90 mmHg or SBP >90 mmHg but requiring bolus infusions and/or vasopressor drugs and/or admission base excess (BE) >−5 mmol/L and/or shock index >1 and/or transfusion requirement of at least 4–6 units of packed RBCs within the first 24 h	97	AE	9/23	NA	53	8
					Non-AE	28/37			
					EPP	19/26			
					Non-EPP	18/34			
					EF	5/14			
					Non-EF	32/46			
				75	AE	12/26	56.7 ± 21.1	40	
					EPP	15/22	58.9 ± 20.2	65	

## Data Availability

The data used to support the findings of this study are included within the article and its supplementary materials.

## Supplementary Materials

Supplementary Materials Supplementary 1 shows the PRISMA checklist. The search strategy we used is detailed in Supplementary 2. Funnel plots assessing publication bias are depicted in Supplementary 3.
