# Supplementary material for: Hemostatic Interventions and All-Cause Mortality in Hemodynamically Unstable Pelvic Fractures: A Systematic Review and Meta-Analysis
Source: Emerg Med Int. 2024 Aug 26;2024:6397444. doi: 10.1155/2024/6397444 (PMC11368555; doi:10.1155/2024/6397444)
Supplement: Supplementary Materials — Supplementary 1 shows the PRISMA checklist. The search strategy we used is detailed in Supplementary 2. Funnel plots assessing publication bias are depicted in Supplementary 3. [file 6397444.f1.zip › Supplementary 2.pdf]

(((Pelvic[Title/Abstract]) OR ("Pelvis"[Mesh])) AND (((((((Fracture[Title/Abstract]) OR (Fractures[Title/Abstract])) OR (Trauma[Title/Abstract])) OR (Traumas[Title/Abstract])) OR (Injury[Title/Abstract])) OR (injuries[Title/Abstract])) OR (Disruption[Title/Abstract])) OR (Disruptions[Title/Abstract]))) AND (((((hemodynamic instability[Title/Abstract]) OR (hemodynamic instabilities[Title/Abstract])) OR (hemodynamically unstable[Title/Abstract])) OR (shock[Title/Abstract])) OR (Hypotension[Title/Abstract]))
