# Supplementary material for: Hemostatic Interventions and All-Cause Mortality in Hemodynamically Unstable Pelvic Fractures: A Systematic Review and Meta-Analysis
Source: Emerg Med Int. 2024 Aug 26;2024:6397444. doi: 10.1155/2024/6397444 (PMC11368555; doi:10.1155/2024/6397444)
Supplement: Supplementary Materials — Supplementary 1 shows the PRISMA checklist. The search strategy we used is detailed in Supplementary 2. Funnel plots assessing publication bias are depicted in Supplementary 3. [file 6397444.f1.zip › Supplementary 3.pdf]

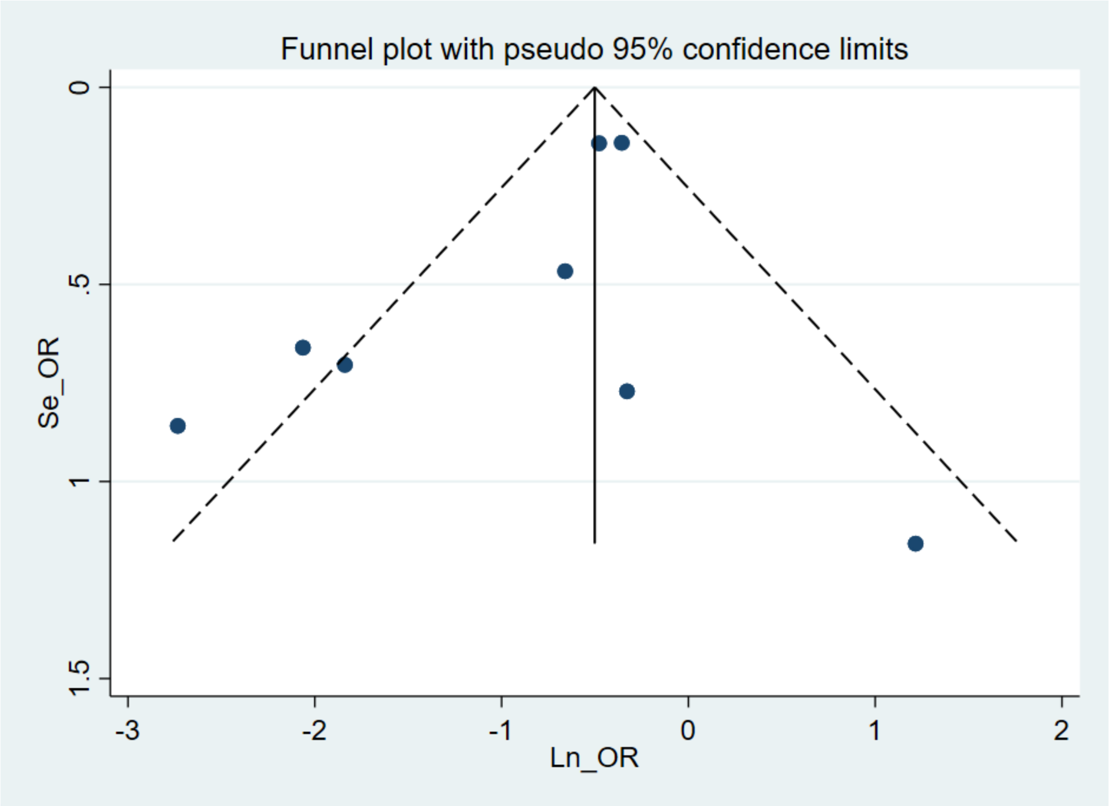
Funnel plot with pseudo 95% confidence limits
0
.5
1
1.5
Se_OR
-3
-2
-1
0
1
2
Ln_OR

a

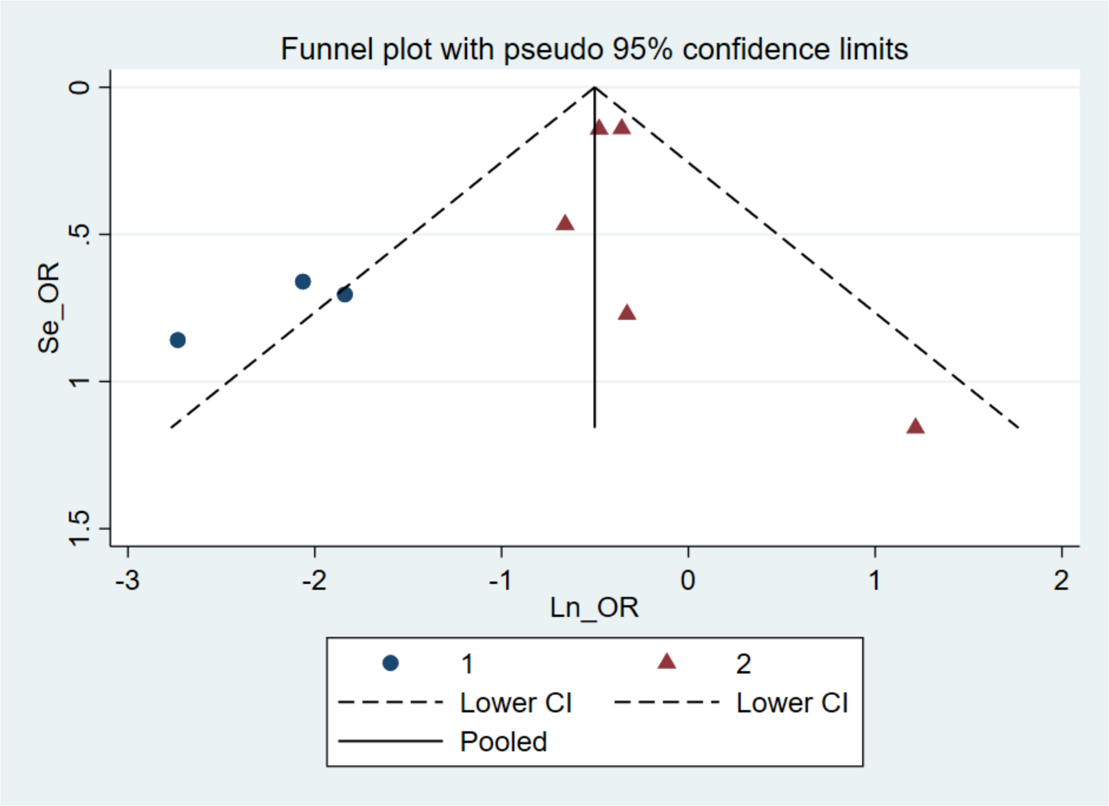
Funnel plot with pseudo 95% confidence limits
0
.5
1
1.5
Se_OR
-3
-2
-1
0
1
2
Ln_OR
1
2
Lower CI
Lower CI
Pooled

b

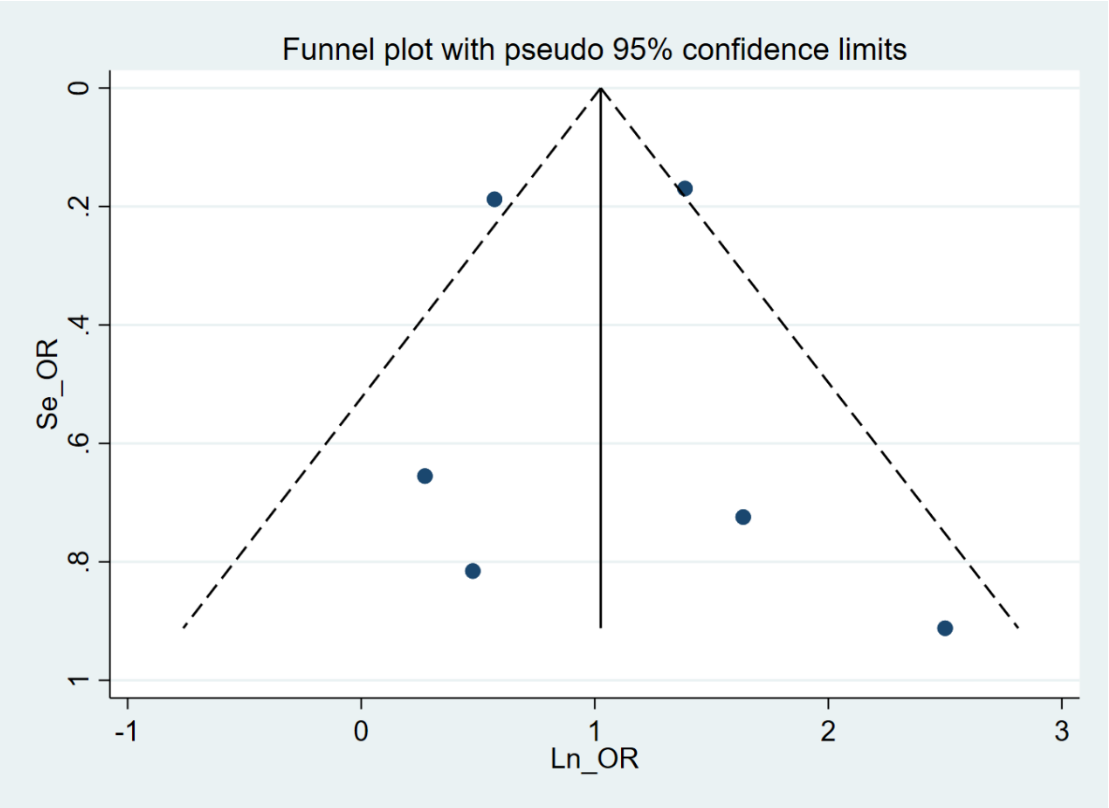
Funnel plot with pseudo 95% confidence limits
Se_OR
0
.2
.4
.6
.8
1
-1
0
1
2
3
Ln_OR

c

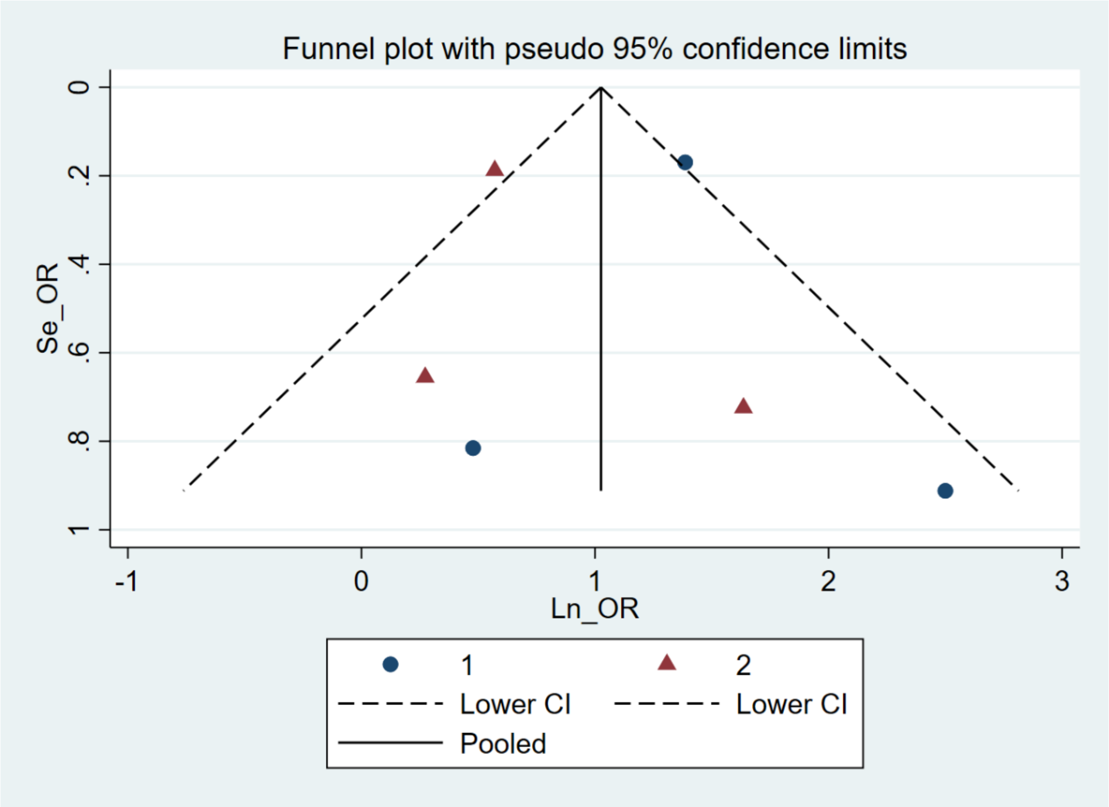
Funnel plot with pseudo 95% confidence limits
Se_OR
0
.2
.4
.6
.8
1
-1
0
1
2
3
Ln_OR
1
2
Lower CI
Lower CI
Pooled

d

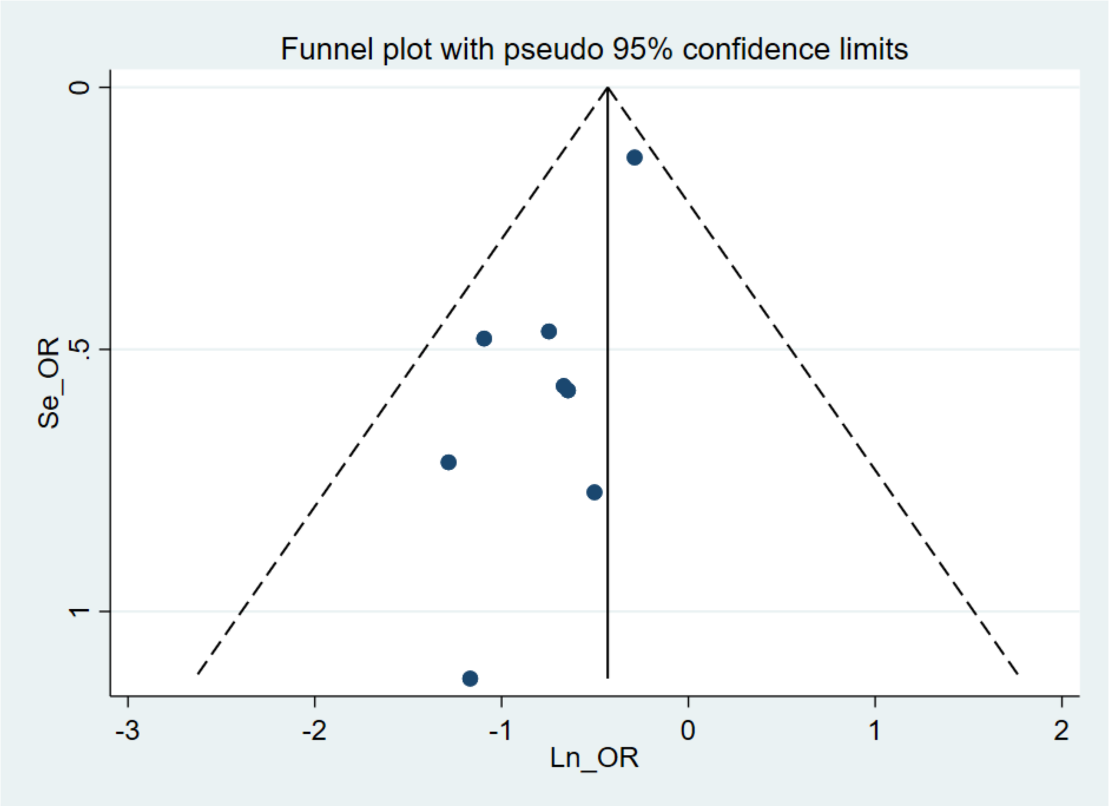
Funnel plot with pseudo 95% confidence limits
0
.5
1
Se_OR
-3
-2
-1
0
1
2
Ln_OR

e

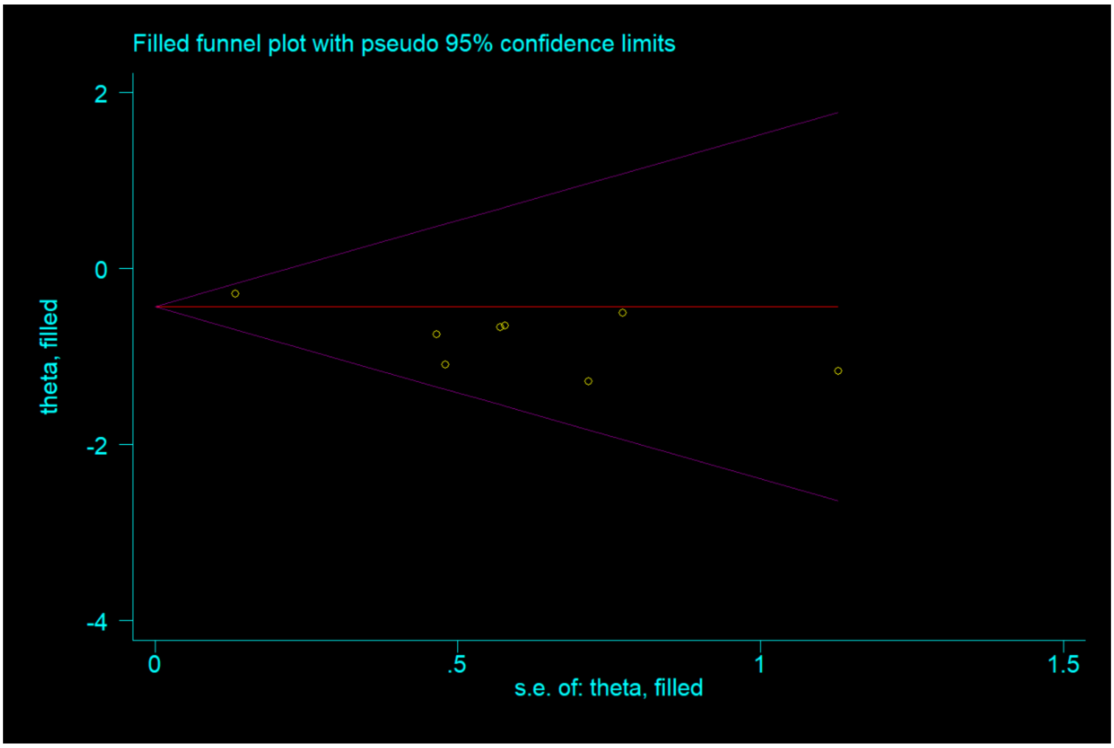
Filled funnel plot with pseudo 95% confidence limits
2
0
-2
-4
theta, filled
0
.5
1
1.5
s.e. of: theta, filled

f

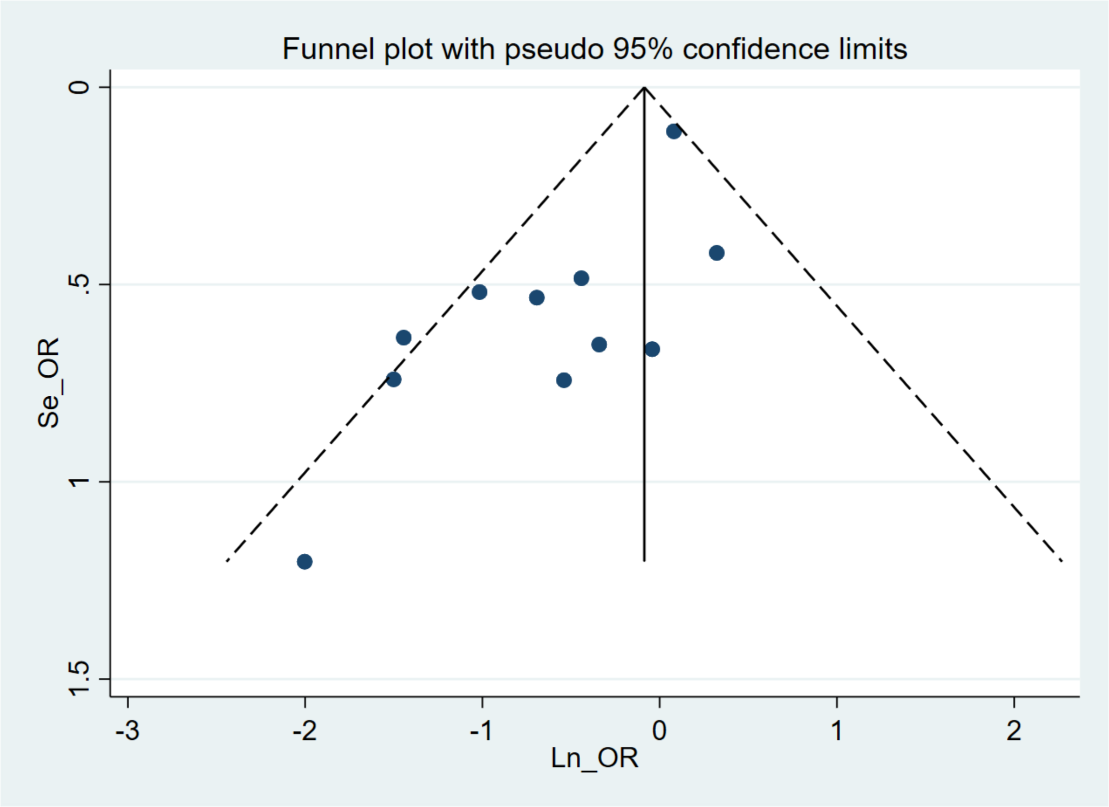
Funnel plot with pseudo 95% confidence limits
0
.5
1
1.5
Se_OR
-3
-2
-1
0
1
2
Ln_OR

g

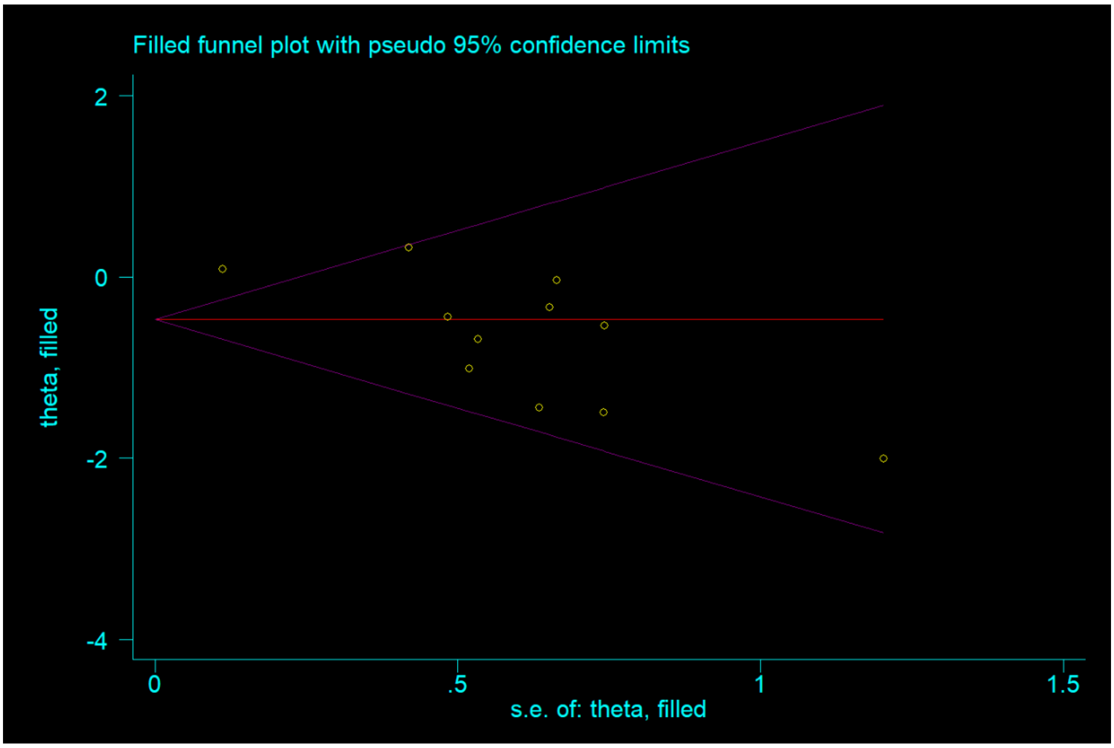
Filled funnel plot with pseudo 95% confidence limits
2
0
-2
-4
theta, filled
0
.5
1
1.5
s.e. of: theta, filled

h

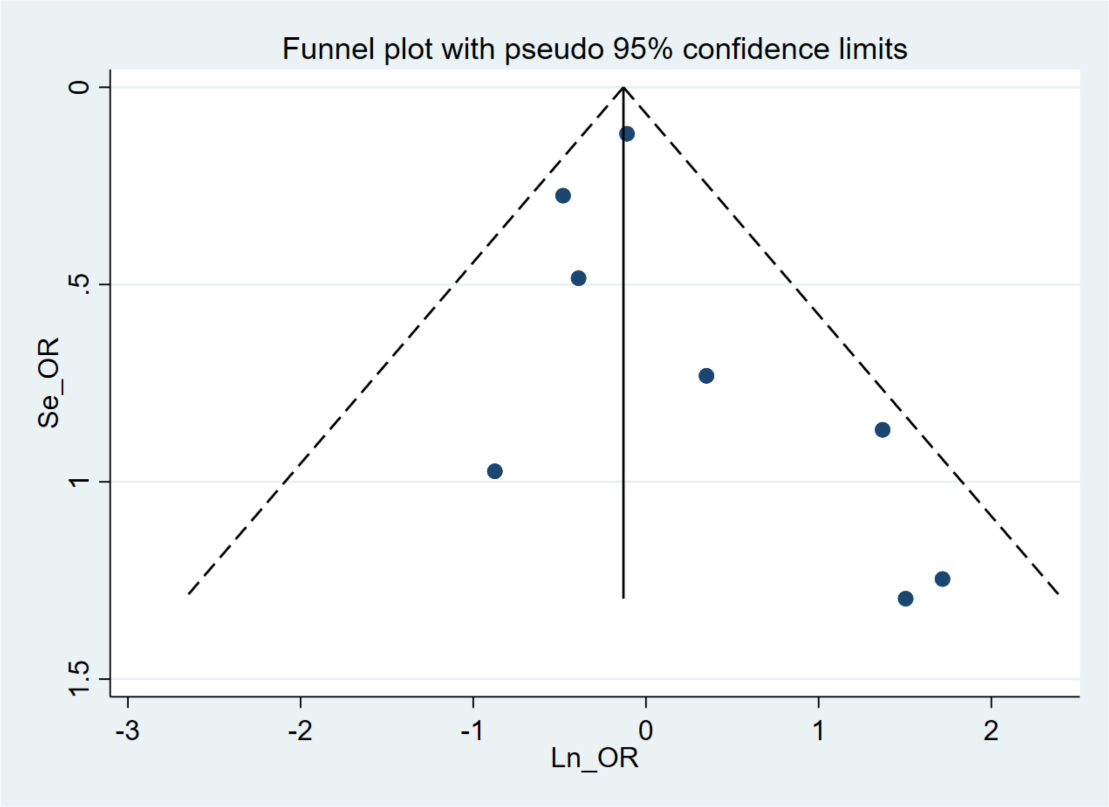

i

Supplementary material 3: Funnel plots assessing publication bias. (a)AE. (b)AE subgroup analysis. (c)REBOA. (d)REBOA subgroup analysis. (e)EF. (f)EF trim and fill. (g)EPP. (h)EPP trim and fill. (i) AE vs EPP.
